# APOOL Is a Cardiolipin-Binding Constituent of the Mitofilin/MINOS Protein Complex Determining Cristae Morphology in Mammalian Mitochondria

**DOI:** 10.1371/journal.pone.0063683

**Published:** 2013-05-21

**Authors:** Tobias A. Weber, Sebastian Koob, Heinrich Heide, Ilka Wittig, Brian Head, Alexander van der Bliek, Ulrich Brandt, Michel Mittelbronn, Andreas S. Reichert

**Affiliations:** 1 Mitochondrial Biology, Buchmann Institute for Molecular Life Sciences, Goethe University, Frankfurt am Main, Germany; 2 Mitochondriale Biologie, Zentrum für Molekulare Medizin, Goethe Universität, Frankfurt am Main, Germany; 3 Molecular Bioenergetics Group, Medical School, Cluster of Excellence Frankfurt Macromolecular Complexes, Goethe University, Frankfurt am Main, Germany; 4 Department of Biological Chemistry, David Geffen School of Medicine, University of California Los Angeles, Los Angeles, California, United States of America; 5 Institute of Neurology, Edinger Institute, Goethe University, Frankfurt am Main, Germany; Auburn University, United States of America

## Abstract

Mitochondrial cristae morphology is highly variable and altered under numerous pathological conditions. The protein complexes involved are largely unknown or only insufficiently characterized. Using complexome profiling we identified apolipoprotein O (APOO) and apolipoprotein O-like protein (APOOL) as putative components of the Mitofilin/MINOS protein complex which was recently implicated in determining cristae morphology. We show that APOOL is a mitochondrial membrane protein facing the intermembrane space. It specifically binds to cardiolipin *in vitro* but not to the precursor lipid phosphatidylglycerol. Overexpression of APOOL led to fragmentation of mitochondria, a reduced basal oxygen consumption rate, and altered cristae morphology. Downregulation of APOOL impaired mitochondrial respiration and caused major alterations in cristae morphology. We further show that APOOL physically interacts with several subunits of the MINOS complex, namely Mitofilin, MINOS1, and SAMM50. We conclude that APOOL is a cardiolipin-binding component of the Mitofilin/MINOS protein complex determining cristae morphology in mammalian mitochondria. Our findings further assign an intracellular role to a member of the apolipoprotein family in mammals.

## Introduction

Mitochondria fulfill a number of essential metabolic functions such as oxidative phosphorylation; synthesis of heme, lipids and amino acids; iron-sulfur biogenesis; and thermogenesis. Furthermore, they are important regulators of apoptosis and represent a major source of reactive oxygen species (ROS). Given these ample tasks it is not surprising that mitochondrial ultrastructure is highly diverse and dynamic [for review see 1]. It depends on cell type and the metabolic state and can change rapidly as e.g. observed during apoptosis [2], [3]. Mitochondria are enclosed by two membranes, the outer membrane (OM) and the inner membrane (IM). The latter can further be subdivided in two subcompartments, the inner boundary membrane (IBM) which is closely apposed to the outer membrane and the cristae membrane (CM) which represents protrusions towards the matrix space. The IBM and the CM exhibit distinct protein compositions which can dynamically change depending on the physiological state of a cell [4], [5], [6]. Moreover, the IBM and the CM are linked by ring- or slot-like tubular connections termed crista junctions (CJs) as revealed by electron microscopy of serial sections [7] or by more recent advances in cryo-electron tomography [for review see 8,9]. These structures are hypothesized to act as diffusion barriers which are critical for establishing distinct subcompartments within mitochondria. They may help to generate proton and/or ADP gradients within the intermembrane space of mitochondria [10], [11]. Furthermore, the diameter of CJs was shown to change dynamically during apoptosis [2], [3], [12]. Only a few proteins have been linked to determine cristae morphology of mitochondria [for review see 1]. Downregulation of Mitofilin, also termed ‘inner membrane protein mitochondrial’, IMMT, or ‘heart muscle protein’, HMP, [13], was shown to impair formation of CJs in mammals [14]. Its ortholog in baker's yeast, Fcj1 (Formation of crista junction protein 1), was shown to be required for CJ formation and to be enriched at CJs [15]. Fcj1 is part of a high molecular weight complex [15] and five novel constituents of this complex have been reported recently [16], [17], [18]. The conserved C-terminus of Fcj1 was further reported to interact with the SAM50/TOB55 complex [19]. This domain and also the coiled-coil domain of Fcj1 are both crucial for CJ formation in mitochondria [19], [20]. Compared to baker's yeast the knowledge about the mammalian Mitofilin/MINOS complex and its constituents is limited. Still, several interaction partners of mammalian Mitofilin were reported including the coiled-coil-helix-coiled-coil-helix domain containing proteins 3 and 6 (CHCHD3 and CHCHD6), Disrupted-in-schizophrenia 1 (DISC1), the sorting and assembly machinery component 50 (SAMM50), Metaxin-1 and -2 (MTX1 and MTX2), and DNAJC11 [16], [21], [22], [23], [24]. A recent report studied the role of CHCHD6 in more detail and showed that CHCHD6 itself is required for normal cristae morphology [25]. Overall, the Mitofilin/MINOS complex and the role of its constituents remains far from being understood. Here we applied complexome profiling of bovine heart mitochondria and identified two novel proteins as putative subunits of this complex: apolipoprotein O (APOO) and the homologous apolipoprotein O-like (APOOL). Classical apolipoproteins are located extracellularly, bind lipids thereby forming lipoproteins, and are essential for the transport of lipids through the circulatory or lymphatic system. APOO has been described as a secreted glycoprotein that is upregulated by diabetes in human heart [26]. Its overexpression was reported to have no impact on plasma HDL levels in apolipoprotein A-l transgenic mice [27]. Nothing was reported on the function of APOOL in mammals so far. A putative homologue of APOO and APOOL from *C. elegans*, Moma-1, has been studied recently and was suggested to be in the same pathway as Mitofilin in determining cristae morphology [28]. In baker's yeast two putative homologs of APOO/APOOL exist [28] which were recently shown to be in Fcj1/MINOS protein complex: Aim37 and Mio27 [16], [17], [18]. Here we report that human APOOL is a mitochondrial membrane protein facing the IMS which interacts with Mitofilin and other components of the MINOS complex. A recombinant version of this protein binds specifically to cardiolipin (diphoshatidylglycerol), a lipid that is found predominantly in the inner mitochondrial membrane. Overexpression as well as downregulation of APOOL resulted in impaired mitochondrial respiration and altered cristae morphology. We propose that APOOL is a non-conventional lipoprotein which has a crucial intracellular role in determining cristae morphology.

## Experimental Procedures

### Plasmids

The human APOOL with C-terminal FLAG was cloned into the pcDNA3.1-vector (gift from Prof. Volker Dötsch, Frankfurt University, Germany) after reverse transcription and PCR amplification of mRNA isolated from cultured HeLa cells (ATCC number CCL-2) with addition of a C-terminal FLAG-tag using the following primers (APOOLfwd: AAA CCG CGG ATG GCG GCC ATC AGG ATG GG and APOOLrev AAA TCT AGA TCA TTT GTC ATC GTC ATC TTT GTA GTC GCT TCT AGT GCT GTA CAT ATC) and SacII and XbaI as restriction sites. The GST-fusion constructs were cloned with a C-terminal HIS-tag into pGEX4T1 vector using the following primers (APOOLGSTfwd AAA GAA TTC ATG GCG GCC ATC AGG ATG GG and APOOLGSTrev AAA GTC GAC TCA ATG GTG ATG GTG ATG GTG GCT TCT AGT GCT GTA CAT ATC) and EcoRI and SalI as restriction sites. For in vivo labeling of mitochondria the plasmid CAG-IRES-mitoDsRed.T4 [29] was used.

### Cell culture

Cells have been cultured in Dulbecco's modified Eagle's media (DMEM) containing 4.5 g/l glucose supplemented with 10% fetal bovine serum, 1% penicillin-streptomycin (100 U/ml) and 1% L-glutamine (200 mM), at 37°C under an atmosphere of 5% CO_2_. Transgene transfection was carried out with Effectene (Qiagen, Germany) according to the manufacturer's instructions.

### Subcellular and submitochondrial localization of APOOL

For subcellular localization of APOOL in HeLa (ATCC number CCL-2) or 143B cells (ATCC number CRL-8303) mitochondria were isolated as described [30]. For the submitochondrial localization of APOOL mitochondria were isolated in the same way but in the absence of protease inhibitors. 50 µg mitochondria (1 µg/ml) in buffer containing 210 mM mannitol, 70 mM sucrose, 1 mM EDTA and 10 mM Hepes pH 7.5 were digested with 10 µg trypsin for 30 min at 4°C without or with increasing amounts of digitonin with a detergent∶protein ratio ranging from 0.01∶1 to 4∶1 (w/w). After 30 min Trypsin inhibitor and 2× Lämmli sample loading buffer was added followed by immediate boiling of the samples and subsequent analysis by SDS-PAGE and immunobloting. The experiments were carried out in triplicate.

### Immunofluorescence microscopy

For immunofluorescence microscopy HeLa cells were plated on 12 mm cover slips, grown overnight and transfected the following day. 24 h after transfection the media was aspirated and cells were fixated with 3.7% formaldehyde in PBS for 10 min at room temperature. Cells were washed four times with PBST (PBS/0.1% Triton-X100) and blocked with 3% (w/v) skim milk in PBST for 30 min at room temperature. Cells were incubated with 100 µl 3% skim milk in PBST containing the corresponding antibody (1∶500) for 1 h at room temperature, followed by three washing steps with PBST and incubation with secondary antibody for 30 min at room temperature. After 3 additional washing steps the nucleus was stained with DAPI diluted in PBST to 1 µg/ml and incubated for 2 min. The cells were washed again four times with PBST and cover slips were mounted onto microscope slides using mounting media and sealed with nail polish. Images were taken with a laser-scanning microscope (Eclipse TE2000e, Nikon), a 1.49 numerical aperture oil immersion lens (APO TIRF 60×, Nikon), and a microscope integrated intermediate magnification of 1.0 to 1.5-fold. The fluorophores DAPI, Alexa488 and DsRed were successively excited with an diode laser, an argon laser and a helium-neon laser (DAPI: 405 nm excitation, 450/15 nm detection; Alexa488: 488 nm excitation, 515/30 nm detection; DsRed: 543 nm excitation, 605/75 nm detection). Background reduction was performed using the software EZ-C1v.3.70 (Nikon) and ImageJ. The experiments were carried out in triplicate.

### Analysis of mitochondrial morphology

HeLa cells were cultured and transiently transfected with the indicated constructs using Effectene (Qiagen, Hilden). To determine the mitochondrial morphology cells were cotransfected with pEGFP-Mito (Clontech Laboratories, USA) or immunostaining was carried out using an antibody against mitochondrial cytochrome *c* coupled to the fluorophor Alexa Fluor 555 (BD Bioscience). For immunostaining cells were fixed on coverslips prior to antibody treatment using 3.7% formaldehyde in DMEM for 10 to 20 min at 37°C, followed by a permeabilization step in ice-cold methanol. Washing was carried out between all incubation steps with PBS for three times.

Mitochondrial morphology was quantified as follows: tubular, more than 70% of the mitochondria show a tubular length of at least 2–3 µm or more in a cell; fragmented, no mitochondrial tubules larger than 2–3 µm in length are present in a cell; intermediate, mitochondrial morphology is neither tubular nor fragmented. For each approach the frequencies of cells with tubular, intermediate, and fragmented mitochondria from three independent experiments (70 to 120 cells were analyzed for each transfection and experiment) were calculated.

### Determination of ROS levels

Superoxide anion levels were measured using the fluorescent dye dihydroethidium (DHE) in a final concentration of 5 µM. Cells were transfected with the indicated constructs and 24 h prior to measurement seeded at a constant density of 1×10^4^ cells per well in a 96-well plate. As control cells were pre-incubated for 4 hours in HBSS a condition known to induce ROS. At the day of measurement DHE was supplemented to the growth medium or to HBSS and the cells were incubated for 10 min at 37°C. The medium was exchanged to PBS and the DHE fluorescence within the cells was measured using a plate reader (Infinite M200Pro, Tecan Deutschland GmbH, Germany) set to 518 nm excitation and 605 nm emission wavelength.

### Recombinant protein expression and protein purification

Recombinant GST-Proteins were expressed in *E. coli* BL21 (RIL) cells. Cells were grown at 37°C in 2× YT media to an optical density_600 nm_ of 0.6 with constant shaking, induced with a final concentration of 0.5 mM IPTG, and grown at 16°C overnight. Cells were collected by centrifugation at 4,000 g for 10 min. All steps were performed at 4°C. Cells were lysed in PBS including protease inhibitors and DNAse using predigestion with lysozyme for 30 min followed by sonification. All following steps were performed using PBS buffer including protease inhibitors (1× Complete w/o EDTA (Roche, Switzerland) and 1 mM PMSF) and 0.01% (w/v) digitonin. The lysates were centrifuged at 40,000 g for 30 min, supernatant 1 was taken and stored overnight (following steps do not apply for GST non-fusion protein until glutathione affinity purification), the pellet was incubated in PBS containing 10% (w/v) N-laurylsarcosyl overnight [31]. Following centrifugation at 40,000 g for 30 min the supernatant 2 was combined with supernatant 1 and digitonin stock solution (20% (w/v) in PBS) to a final concentration of 4% (w/v) digitonin and 1% (w/v) N-laurylsarcosyl and incubated for 30 min. This mixture was then incubated with an appropriate volume of equilibrated NiNTA resin, imidazole (20 mM) and 0.01% (w/v) digitonin, following washing with 60 mM imidazole (>10 resin volumes). The proteins were eluted with 250 mM imidazole. The elutions were diluted 1∶5 and then applied on a glutathione resin (in case of GST non-fusion supernatant 1 was diluted 1∶3 and applied). Resin was washed (>10 resin volumes) and subsequent elution followed; the elution buffer contained 10 mM glutathione.

### Lipid binding assay

Membrane lipid strips™ (Echelon biosciences) were used in accordance to the manufacturer's instructions. Blocking and incubation was performed using PBS containing 1% (w/v) skim milk and 0.01% (w/v) digitonin. Protein elution fractions were diluted 1∶5 in blocking solution, the following procedures accorded to the manufacturer's instructions. Washing buffer was PBS containing 0.05% (v/v) Tween-20. Bound proteins were detected using antibodies raised against GST and secondary donkey-anti-goat-HRP-coupled antibodies (Santa Cruz, USA) using chemiluminescence. The experiments were carried out in triplicate.

### SDS-PAGE and Western blot analysis

Equal protein amounts of total cell extracts were separated by SDS-PAGE and analyzed by immunoblotting using the following antibodies recognizing: β-actin (Cell Signaling, #4967), mtHSP60 (Cell Signaling, #4870), HSP40 (abcam, ab69402), human APOOL (Atlas Antibodies, HPA000612), TOM20 (Atlas Antibodies, HPA011562), human Mitofilin (Pineda, Berlin, polyclonal antibody raised in rabbits against CTDHPEIGEGKPTPALSEEAS), human MINOS1 (Pineda, Berlin, polyclonal antibody raised in rabbits against CQHDFQAPYLLHGKYVK), human OPA1 (Pineda, Berlin, polyclonal antibody raised in rabbits against the C-terminus of human OPA1 using the synthetic peptide CDLKKVREIQEKLDAFIEALHQEK), DRP1 (BD Transduction Laboratories, #611112), FLAG-Tag (Gilbertsville, USA or Sigma-Aldrich, s3165), GST (Millipore, AB3282). Quantification of Western blots was done using the ChemiDoc XRS+ system and Quantity One software (BioRad, Germany).

### Co-immunoprecipitation

For immunoprecipitation of endogenous Mitofilin or APOOL mitochondria isolated from HeLa cells (250 µg total mitochondrial protein) were solubilized using a detergent∶protein ratio of 4∶1 at 4°C followed by a centrifugation at 20.000 g for 15 min at 4°C. The supernatant was then incubated at 4°C with 1 µg antibody (APOOL, Mitofilin or preimmunserum) for 1 h followed by incubation with equilibrated protein A beads for 1 h at 4°C. All following centrifugation steps were carried out at 500 g for 1 min at 4°C. The beads were washed five times with PBS containing 0.01% digitonin. Proteins were eluted with 0.1 M citric acid with subsequent addition of 2× Lämmli sample loading buffer and boiling of samples. Analysis was done by SDS-PAGE and subsequent immunoblotting. The experiments were carried out in triplicate.

### Determination of oxygen consumption rate (OCR)

A Seahorse Bioscience XF24-3 Extracellular Flux Analyzer was used to measure the O_2_ consumption rate in medium surrounding the adherent cells cultured in a XF24 cell culture plate (Seahorse Bioscience). Hela cells were transfected twice with the corresponding DNA constructs and seeded 24 h prior to measurement at a constant density of 2×10^4^ cells per well in a XF 24-well-plate.

The following day additional medium was added to the 24-well-plate to reach the required assay volume of 650 µl and cells were pre-incubated at 37°C without CO_2_ atmosphere. The uncoupler carbonyl–cyanide-(trifluoromethoxy)-phenylhydrazone (FCCP) was preloaded in the reagent delivery chambers of the sensor cartridge and after equilibration of the sensor cartridge the measurement was started. DMSO as a control followed by increasing FCCP concentrations were pneumatically injected into the wells to reach the indicated final concentration of 10 µM, 20 µM, and 40 µM. For each condition at least four independent wells were analyzed. Basal respiration was calculated by using the Seahorse XF24 Extracellular Analyzer software 1.7. The relative OCR induction by FCCP was calculated by setting the respective DMSO control as 100%. Background corrections and normalization were done using the Seahorse XF24 Extracellular Analyzer software 1.7.

### Complexome profiling

For complexome profiling bovine heart mitochondria were isolated as described [32] and solubilized with digitonin at a detergent/protein ratio of 6 g/g in 50 mM imidazole/HCl (pH 7.0), 50 mM NaCl, 2 mM 6-aminohexanoic acid, 1 mM EDTA [33]. After a clarifying spin (22,000 g, 20 min 4°C) solubilized mitochondrial proteins (0.2 mg total protein) were applied to a 5%T/20%C - 9%T/3%C [see 34] acrylamide gradient large pore blue native electrophoresis (LP-BNE) gel with an 5%T/25%C sample gel as described [35]. 1D gel lanes were fixed in 50% methanol and 10% acetic acid, stained with Coomassie and cut into 52 equally sized slices (#1 to #52 from low to high molecular weight). In-gel tryptic digests were done in perforated well plates, essentially following the protocol by Collins *et al.* [36] and described in Heide *et al.* [37]. Tryptic peptides were subjected to LC-MS/MS analysis in an Orbitrap XL mass spectrometer (Thermo) with an Agilent1200 nano-HPLC at the front end as described in Heide *et al.* [37]. Extraction of peak lists from RAW files was done using Extract-MSn (Bioworks-Package, Thermo Scientific) and MS/MS spectra searched by matching against the *Bos taurus* un-reviewed protein database (22,080 sequences) downloaded from UniProt (Uniprot Consortium, 2010) with Mascot server 2.2 using following settings: precursor mass tolerance 10 ppm, carbamidomethylation of cysteine as fixed and oxidation of methionine as optional modifications, full tryptic cleavage with two missed cleavages. Mascot peptide search results were filtered by Individual ions scores>20 (scores indicating identity or extensive homology (p<0.05)) and used as input for label free quantification into the MSQuant program version 2.0 [38]. XIC values (extracted ion chromatogram) for individual peptides of a given protein and gel spot were summed and used to construct quantitative migration profiles of proteins though the gel lane. Contaminations like keratins, hemoglobins and trypsin were removed from the list. Protein migration profiles were normalized between 0 and 1 and hierarchically clustered using Cluster 3.0 software [39], [40]. Distance measures based on Pearson correlation coefficient (uncentered) and single linkage clustering were used. Clustered profiles were visualized using MS Excel software. Mass calibration was performed using the reported apparent molecular masses of complex II (123 kDa), complex III (482 kDa), complex IV (205 kDa), complex V_monomer_ (597 kDa), supercomplex I/III_2_ (1500 kDa), and supercomplex I/III_2_/IV (1700 kDa) of bovine heart mitochondria as described in [41] resulting in a separation range from roughly ∼70 kDa to 15,000 kDa of the LP-BNE gel.

### Electron microscopy

HeLa or 143B cells were fixed for 45 min using 2.5% (v/v) glutaraldehyde buffered in cacodylate buffer pH 7.4 and centrifuged to form a cell pellet. The embedding procedure comprised fixation in 1% osmium tetroxide, dehydration in a graded ethanol series intermingled by an incubation step with uranyl acetate (between the 50% and 90% ethanol step) and finally rinsing in propylene oxide. The specimens were then embedded in epoxy resins that polymerized for 16 h at 60°C. After embedding, first semithin sections (0.5 µm) were cut using an ultramicrotome (Leica Ultracut UCT, Deerfield, IL, USA) with a diamond knife. Sections were stained with Toluidine blue, placed on glass slides and examined by light microscopy to select appropriate areas for ultrathin preparation. Ultrathin sections (50–70 nm) were cut again using an ultramicrotome. Sections were mounted on copper grids and contrasted with uranyl acetate for 2–3 h at 42°C and lead citrate for 20 min at room temperature. These samples were imaged and digital pictures were taken with a FEI Tecnai G2 Spirit Biotwin TEM (Hillsboro, OR) at an operating voltage of 120 kV.

### RNA interference

Downregulation of target proteins was done using the Block-iT™ POL II miR RNAi Expression Vector Kit with EmGFP (Invitrogen) according to the manufacturer's instructions. For efficient downregulation of APOOL the miRNA constructs were transfected a second time 48 to 72 hours after the first transfection. Downregulation was analyzed by SDS-PAGE and immunoblotting. The experiments were carried out in duplicate. Primer-sequences used for APOOL downregulation: forward: TGC TGT TTG CTG GCT TGT AGC ATA TAG TTT TGG CCA CTG ACT GAC TAT ATG CTA AGC CAG CAA A; reverse: CCT GTT TGC TGG CTT AGC ATA TAG TCA GTC AGT GGC CAA AAC TAT ATG CTA CAA GCC AGC AAA C

### Statistical analysis of mitochondrial ultrastructure

For APOOL-FLAG overexpression and the corresponding control we determined the frequencies of mitochondrial sections exhibiting twirled and circular cristae membranes. For APOOL miRNA downregulation and the corresponding control we determined the frequencies of mitochondrial sections containing concentric, interconnected and/or branched cristae. Nominal scaled response variable (mitochondria exhibiting altered cristae morphology: yes/no) and nominal explanatory variable (APOOL up- or downregulation) were statistically analyzed using contingency analysis and subsequently tested by the likelihood-ratio test. A significance level of α = 0.05 was chosen for statistical testing. Statistical analysis was performed using JMP 8.0 software (SAS, Cary, NC, USA).

For comparison of the mitochondrial area per section of different conditions as evaluated by electron microscopy, the area of 25 to 26 mitochondria was determined for each condition. Statistical analyses were performed by using an unpaired two-tailed student's t-test for each pair comparison.

### Bioinformatic analysis of APOOL

The protein sequence of human APOOL and APOO and selected orthologs was analyzed with the bioinformatic analysis software Mitoprot [42] for prediction of N-terminal mitochondrial target sequences and DAS [43] for prediction of transmembrane helices.

## Results

### Complexome profiling reveals APOOL and APOO as putative constituents of the Mitofilin/MINOS protein complex

**Figure 1:**
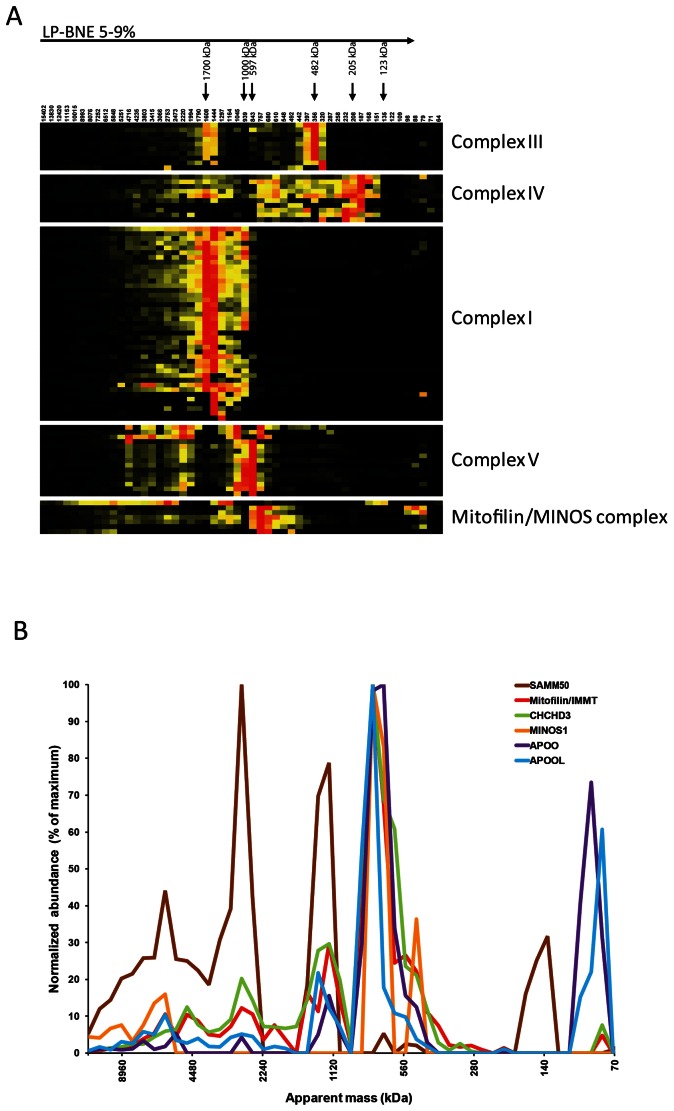
Complexome profiling of bovine heart mitochondria. **A**, Bovine heart mitochondria were solubilized in digitonin, separated by 5–9% large pore blue native gel electrophoresis (LP-BNE), 52 gel slices/fractions were obtained for quantitative mass spectrometry, and hierarchical clustering using size distribution profiles of identified proteins was performed. Selected complexes involved in oxidative phosphorylation (complex I, III, IV, and V) and the Mitofilin/MINOS complex are shown. The known masses of complex II (123 kDa), complex III (482 kDa), complex IV (205 kDa), complex V_monomer_ (597 kDa), of complex I (1000 kDa), and of supercomplex I/III_2_/IV (1700 kDa) of bovine heart mitochondria are indicated and were used for mass calibration. Calculated apparent masses in kDa are indicated on top of each fraction. Protein abundance is colored from high (red) to medium (yellow) to low (black). **B**, Size distribution profiles of known and putative constituents of the Mitofilin/MINOS complex are shown. The normalized abundance for each protein at a given apparent molecular mass (kDa) is given as percentage of the maximal value obtained in any of the 52 gel slices/fractions.

To identify novel factors regulating cristae morphology we aimed to identify novel constituents of the Mitofilin/MINOS protein complex. For that we applied a recently reported method termed complexome profiling [37]. Purified bovine heart mitochondria were solubilized using digitonin as detergent and native protein complexes were separated by large-pore blue native gel electrophoresis [35]. 52 gel slices were analyzed by quantitative mass spectrometry and for each identified protein a profile representing the relative abundance at a given size in the large-pore gel was computed. Hierarchical clustering of those profiles revealed protein clusters with similar distribution profiles. As reported previously for human mitochondria [37] and as demonstrated here for bovine heart mitochondria this method robustly allows the detection of well-known mitochondrial protein complexes such as complex I, III, IV, and V involved in oxidative phosphorylation and its constituents (Fig. 1A). This approach further revealed the Mitofilin/MINOS protein complex as two known constituents of this complex, namely CHCHD3, and MINOS1, showed a remarkable similar complex size distribution as Mitofilin (Fig. 1B). Also SAMM50 was clustered closely to this complex as it showed a partially overlapping complex size distribution (Fig. 1B) consistent with the reported interaction between SAMM50 and Mitofilin [22]. In addition, we noted that apolipoprotein O (APOO) and apolipoprotein O-like (APOOL) clustered with Mitofilin, CHCHD3, MINOS1 and also to a considerable extent with SAMM50. These observations suggest that APOO and APOOL are present in mitochondria as constituents of the known Mitofilin/MINOS protein complex. This is also remarkable as these two proteins which belong to the protein family of lipoproteins have not been localized to mitochondria before.

### APOOL is a mitochondrial membrane protein facing the intermembrane space

**Figure 2:**
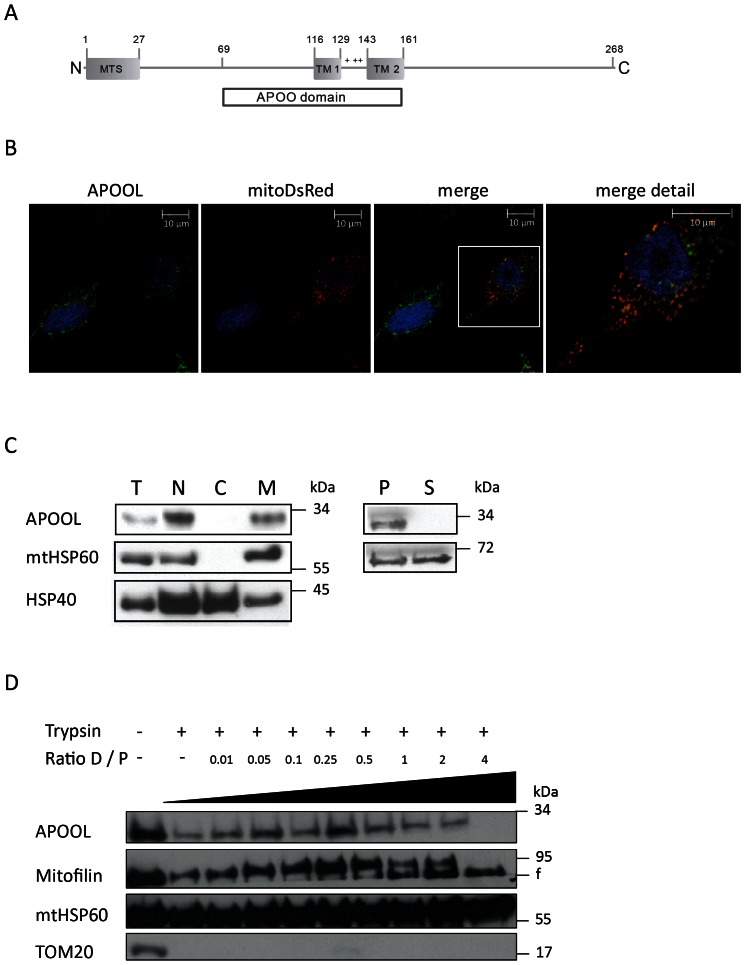
APOOL is a mitochondrial membrane protein exposing major parts to the intermembrane space. **A**, Schematic representation of human APOOL. The conserved APOO domain, the predicted mitochondrial targeting sequence (MTS), and two putative transmembrane helices connected by a positively charged stretch of amino acids are indicated. **B**, Immunofluorescence microscopy of HeLa cells showing the mitochondrial localization of endogenous APOOL (green), mitoDsRed (red), and nuclear DAPI staining (blue). **C**, Left panel, western blot analysis of the subcellular fractionation of osteosarcoma cells (143B) showing that APOOL is enriched in the mitochondrial fraction. HSP40 represents a cytosolic marker and mtHSP60 a mitochondrial marker. T, total cells, N, nuclear fraction, C, cytosolic fraction, M, mitochondrial fraction. Right panel, western blot analysis of alkaline carbonate extraction experiment using the mitochondrial fraction. MtHSP60 is shown as a control. P, pellet, S, supernatant. **D**, Submitochondrial localization of APOOL. Western blot analysis of protease protection assay using isolated mitochondria. The digitonin to protein (D/P) ratio was increased in several steps from 0 to 4 for the successive solubilization of the outer and, at higher digitonin concentrations, of the inner mitochondrial membrane. Trypsin was added for 30 min where indicated. MtHSP60 is shown as a mitochondrial matrix protein, TOM20 as a mitochondrial outer membrane protein and Mitofilin as a mitochondrial inner membrane protein. A tryptic fragment (f) of Mitofilin is depicted.

To corroborate these results we determined the subcellular localization of endogenous APOOL. We decided to focus on the role of APOOL as for this protein no published data was available so far. Bioinformatic analyses revealed that APOOL contains two hydrophobic stretches which are predicted to represent transmembrane helices (Fig. 2A). The presence of two hydrophobic segments is conserved in other members of the APOO protein family such as APOO (*H. sapiens*); APOO and APOOL (*Mus musculus*), Moma-1 (*C. elegans*), Aim37 and Mio27 (*S. cerevisiae*) (data not shown and [28]). These segments are located within the predicted APOO domain (Pfam domain PF09769). Furthermore, APOOL appears to contain an N-terminal mitochondrial targeting sequence (MTS) albeit a cleavage site for the mitochondrial processing peptidase was not predicted. To analyze the subcellular location of APOOL we used HeLa cells expressing mitochondrial mitoDsRed protein and performed immunofluorescence microscopy using antibodies raised against APOOL. We observed a clear colocalization of the APOOL corresponding signal with the mitochondrial marker mitoDsRed indicating that endogenous APOOL is a mitochondrial protein (Fig. 2B). Next we performed a biochemical subcellular fractionation of 143B osteosarcoma cells and cytosolic, nuclear and mitochondrial fractions were generated and analyzed by western blot using indicated markers (Fig. 2C). Endogenous APOOL was found to be entirely absent in the cytosolic fraction and instead was detected in the mitochondrial fraction. APOOL behaved identical to the mitochondrial marker protein mtHSP60 and clearly distinct to the cytosolic marker HSP40. The latter protein showed a relatively weak signal in the mitochondrial fraction which we attribute to unspecific binding of the chaperone HSP40 to hydrophobic mitochondrial outer membrane proteins. The fact that all proteins are detected in the crude nuclear fraction is due to an incomplete lysis of cells which consequently appear in the same fraction as crude nuclei. The data obtained by fluorescence microscopy and subcellular fractionation allow us to conclude that APOOL is a mitochondrial protein.

To elucidate whether APOOL is a soluble or a membrane-bound protein we performed alkaline carbonate extraction using isolated mitochondria from 143B cells. APOOL was found to remain fully in the membrane fraction whereas the chaperone mtHsp60 was released efficiently, yet not completely, to the soluble fraction (Fig. 2C). The incomplete release of mtHsp60 is attributed to its known affinity to hydrophobic membrane proteins. We conclude that APOOL is an integral or strongly membrane-associated protein.

To determine the submitochondrial localization of APOOL a protease protection assay was carried out. Isolated mitochondria were incubated in the presence of trypsin with increasing amounts of digitonin or as controls in the absence of trypsin and/or digitonin. APOOL showed a protease resistance under these conditions that was very similar to the intermembrane space marker Mitofilin. Both proteins were fully degraded only at high concentrations of digitonin (see most right lane, Fig. 2D). The fact that both proteins were already partially digested in the absence of digitonin is attributed to a partial opening of the mitochondrial outer membrane during sample preparation. The signal of the outer membrane marker TOM20 was only detectable in the absence of trypsin whereas the matrix marker mtHsp60 was not accessible to trypsin digestion at any digitonin concentration used. Taken together, we conclude that APOOL is located in the intermembrane space consistent with the proposed location of the Mitofilin/MINOS complex.

### APOOL is a cardiolipin binding protein

**Figure 3:**
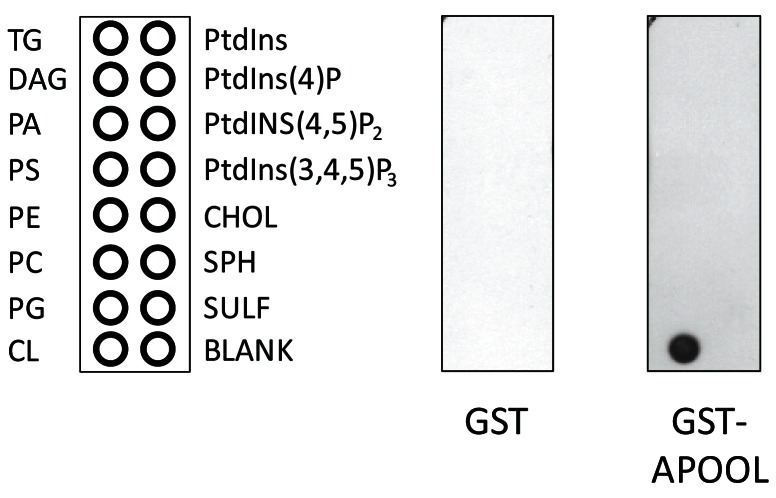
APOOL is a cardiolipin-binding protein. Lipid binding assay using recombinantly expressed and affinity purified GST-APOOL and GST. DAG, diacylglycerol; PA, phosphatidic acid; PS, phosphatidylserine; PE, phosphatidylethanolamine; PC, phosphatidylcholine; PG, phosphatidylglycerol; PI, phosphatidylinositol.

To further characterize APOOL we expressed it as a GST-APOOL-HIS_6_ fusion protein, affinity purified the protein, and performed a lipid binding assay. As control we used a GST non-fusion protein. GST-APOOL-HIS_6_ was found to bind specifically to cardiolipin (CL) but not to any of the other lipids tested, notably not even to phosphatidylglycerol the precursor of cardiolipin (Fig. 3). The control GST non-fusion protein did not bind to any lipid. These findings suggest that the function of APOOL is linked to its ability to bind the mitochondrial lipid cardiolipin.

### Overexpression of APOOL results in mitochondrial fragmentation, reduced oxygen consumption, and altered cristae morphology

**Figure 4:**
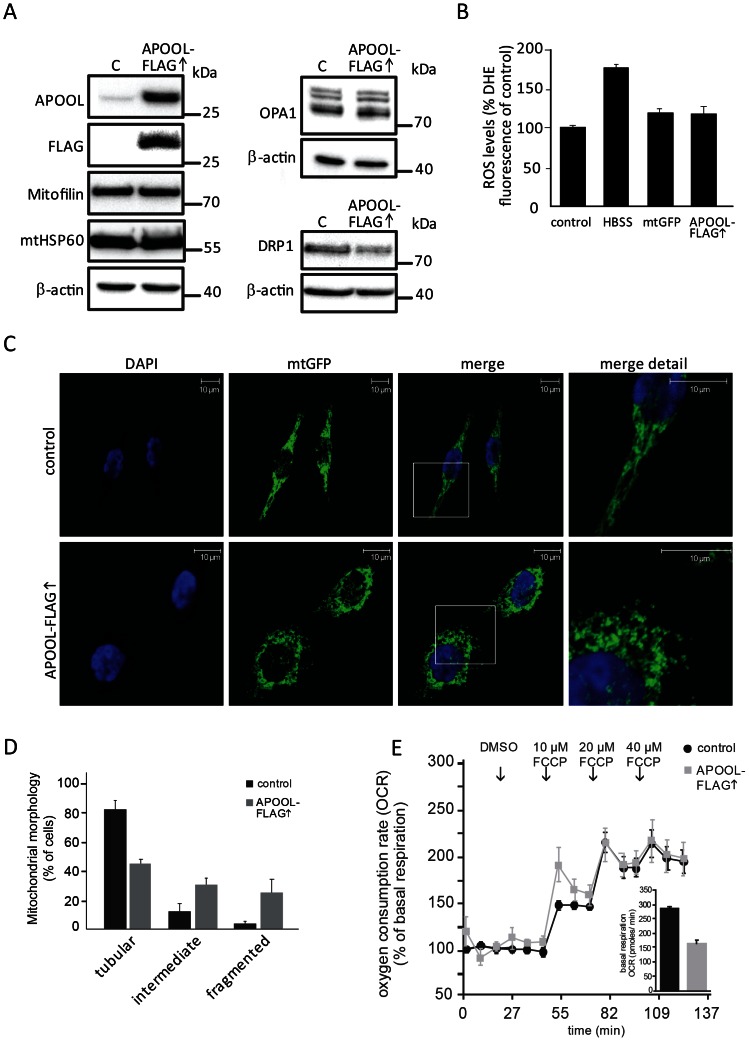
Overexpression of APOOL alters mitochondrial morphology. **A**, Western blot analysis of total cell extracts of HeLa cells after transfection with plasmid DNA encoding APOOL-FLAG or after mock transfection (control) with indicated antibodies. Equal amounts of protein were loaded. **B**, Analysis of ROS formation. HeLa cells have been analyzed by assessing the formation of DHE derived fluorescence. HeLa cells overexpressing APOOL-FLAG or mtGFP (mtGFP), or untreated cells (control), or cells starved by incubation for 4 h in HBSS (HBSS) prior to DHE treatment have been analyzed. The results are shown as mean ± s.d. (n = 4). **C**, Immunofluorescence microscopy of HeLa cells overexpressing mtGFP (control) or mtGFP and APOOL-FLAG. Nuclear DAPI staining is shown in blue. 1^st^ panel, DAPI (blue); 2^nd^ panel, mtGFP (green); 3^rd^ panel, merge; 4^th^ panel, blow-up of indicated white box in 3^rd^ panel. **D**, Quantification of mitochondrial morphology. Mitochondrial morphology of the experiment described in panel C was determined from three independent experiments. 70 to 120 cells were analyzed for each experiment and results are shown as mean ± s.d. (n = 3). Quantification was performed using three categories: tubular, intermediate and fragmented (see methods section).**E**, Determination of oxygen consumption rate (OCR) relative to DMSO and basal respiration (inlay). Increasing FCCP concentrations have been applied at indicated time points. For APOOL and mtGFP overexpression HeLa cells were transfected twice prior to analysis.

**Figure 5:**
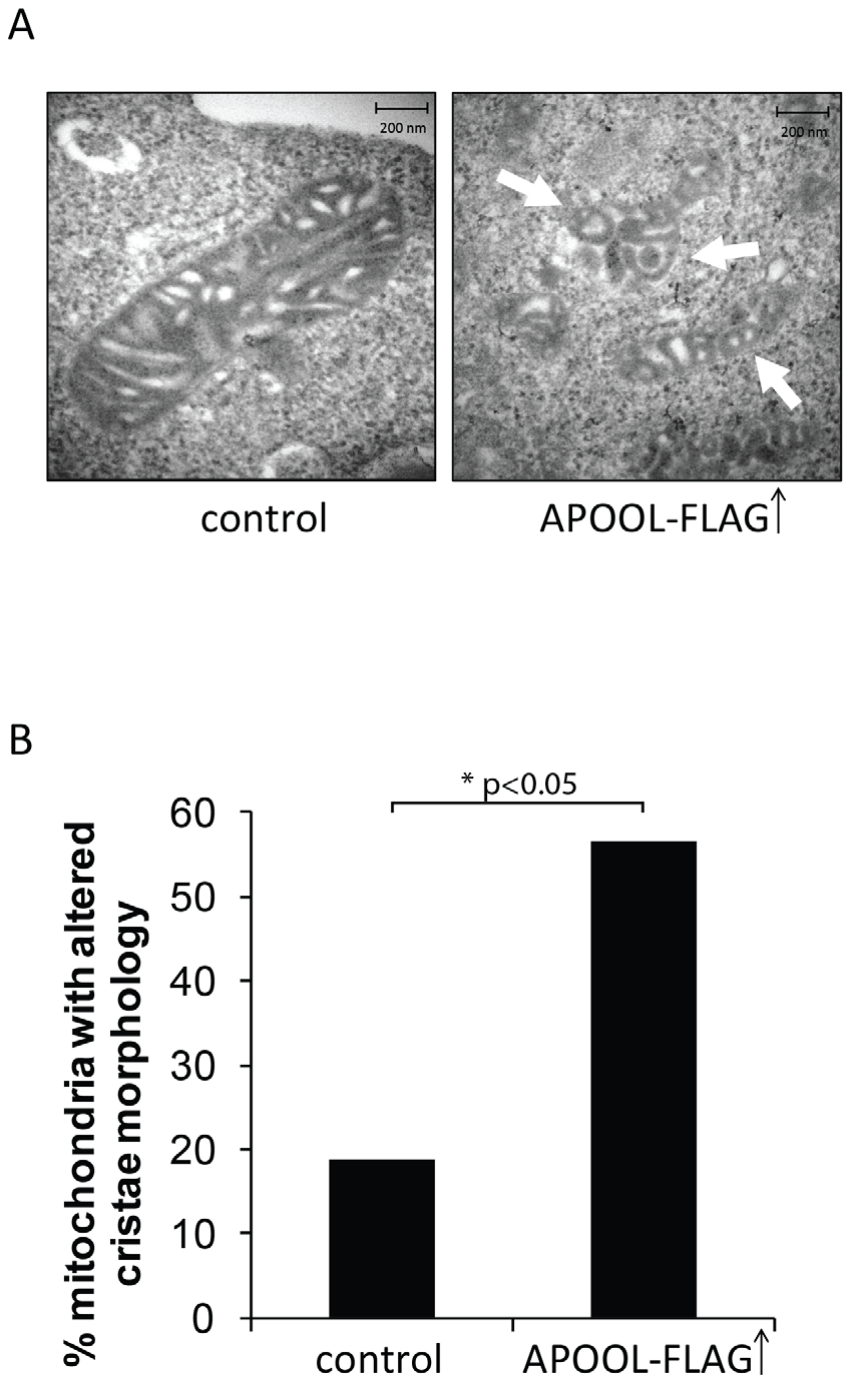
Overexpression of APOOL causes alteration of cristae morphology. **A**, Electron micrographs of 143B cells after transfection with plasmid DNA encoding APOOL-FLAG or mtGFP (control). White arrows indicate twirled and circular cristae membranes. **B**, Quantification of mitochondrial ultrastructure. The frequency of mitochondria showing twirled or circular cristae for APOOL-FLAG overexpression (n = 23) and control (n = 16) were determined. Differences in the amount of mitochondria showing altered cristae morphology were statistically assessed using contingency table analysis followed by the likelihood-ratio test.

Next we analyzed the effects of overexpressing APOOL. For that FLAG-tagged APOOL was transiently overexpressed in HeLa or 143B cells. Overexpression of APOOL-FLAG was confirmed by western blot analysis using an antibody against the FLAG-tag as well as against endogenous APOOL (Fig. 4A, left panel). Overexpression of APOOL-FLAG did not appear to affect the protein levels of Mitofilin, mtHSP60, or OPA1 in total cell extracts (Fig. 4A, left panel and top right panel). However, the level of the mitochondrial fission factor DRP1 was moderately reduced (Fig. 4A, bottom right panel). Overexpression of APOOL did not lead to a significant increase in ROS formation neither when compared to the corresponding control expressing only mtGFP or to untransfected cells (Fig. 4B). As a positive control we starved cells for 4 hours in HBSS which is known to lead to increased ROS formation [44], [45]. To confirm that overexpression or adding a FLAG-tag did not alter the cellular localization of APOOL we coexpressed mitochondrial GFP (mtGFP) and performed immunofluorescence microscopy. APOOL-FLAG was clearly co-localized with the mitochondrial marker mtGFP (data not shown) demonstrating that the mitochondrial localization of APOOL-FLAG is preserved. Further, we noted that overexpression of APOOL-FLAG led to an increased fragmentation of mitochondria (Fig. 4CD). Based on our data described above we can exclude that this observation can be explained by reduced levels or increased proteolytic processing of the fusion factor OPA1 [30], by increased levels of the fission factor DRP1, or by excessive ROS production (Fig. 4AB). It could represent an indirect consequence of overexpression, however, we regard this as unlikely as overexpression of mitochondrial GFP (mtFGP) did not result in mitochondrial fragmentation (Fig. 4CD). To further investigate the influence of overexpression of APOOL-FLAG on mitochondrial function we determined the basal mitochondrial oxygen consumption rate (OCR) and the extent to which this rate is increased by dissipating the mitochondrial membrane potential. Cells expressing APOOL-FLAG showed a considerably lower basal OCR in comparison to control cells (Fig. 4E inlay) indicating that APOOL-FLAG overexpression impairs bioenergetic functions of mitochondria. This observation could well contribute to our observation that mitochondria become fragmented. Next we determined the relative increase of the OCR after applying increasing concentrations of the uncoupler FCCP. Uncoupling with 10 µM FCCP led to an increase of the oxygen consumption rate which was moderately higher in APOOL-FLAG overexpressing cells compared to control cells (Fig. 4E). However, when the concentration was raised to 20 µM or 40 µM FCCP no significant differences were detectable. We conclude that APOOL-FLAG overexpression impairs basal mitochondrial respiration but has no gross effect on the relative increase in mitochondrial respiration induced by FCCP. Next we determined whether APOOL-FLAG overexpression affects mitochondrial ultrastructure by electron microscopy. In electron micrographs of chemically fixed cells overexpressing APOOL-FLAG we observed abnormal cristae morphology characterized by twirled and/or circular cristae membranes (Fig. 5A). The frequency of altered cristae morphology was significantly increased upon overexpression of APOOL-FLAG as compared to when mtGFP was overexpressed (Fig. 5B). We conclude that APOOL overexpression alters cristae morphology possibly resulting in overall alterations of mitochondrial morphology.

### Downregulation of APOOL impairs mitochondrial function and alters cristae morphology

**Figure 6:**
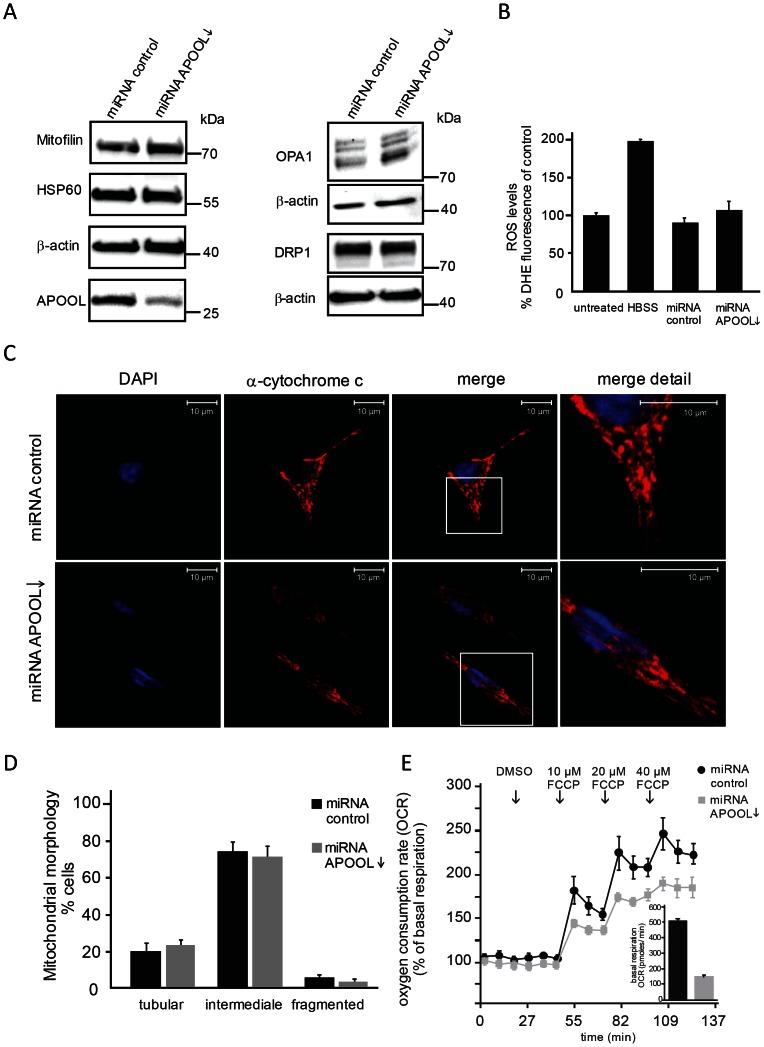
Downregulation of APOOL leads to a decreased basal mitochondrial oxygen consumption rate. **A**, Western Blot analysis of total cell extracts of HeLa cells after transfection with plasmid DNA encoding miRNA against APOOL or control miRNA. **B**, Analysis of ROS formation. HeLa cells have been analyzed by assessing the formation of DHE derived fluorescence. HeLa cells showing low levels of APOOL as well as their corresponding transfection control, untreated cells, or cells starved by incubation for 4 h in HBSS (HBSS) prior to DHE treatment have been analyzed. The results are shown as mean ± s.d. (n = 4). **C**, Immunofluorescence microscopy of HeLa cells stained with an antibody against mitochondrial cytochrome *c*. Nuclear DAPI staining is shown in blue. 1^st^ panel, DAPI (blue); 2^nd^ panel, α-cytochrome *c* (red); 3^rd^ panel, merge; 4^th^ panel, blow-up of indicated white box in 3^rd^ panel. **D**, Quantification of mitochondrial morphology. Mitochondrial morphology of the experiment described in panel C was determined from three independent experiments. 70 to 120 cells were analyzed for each experiment and results are shown as mean ± s.d. (n = 3). Quantification was performed using three categories: tubular, intermediate and fragmented (see methods section). **E**, Determination of oxygen consumption rate (OCR) relative to DMSO and basal respiration (inlay). Increasing FCCP concentrations have been applied at indicated time points. For APOOL downregulation and miRNA control, HeLa cells were transfected twice prior to analysis.

**Figure 7:**
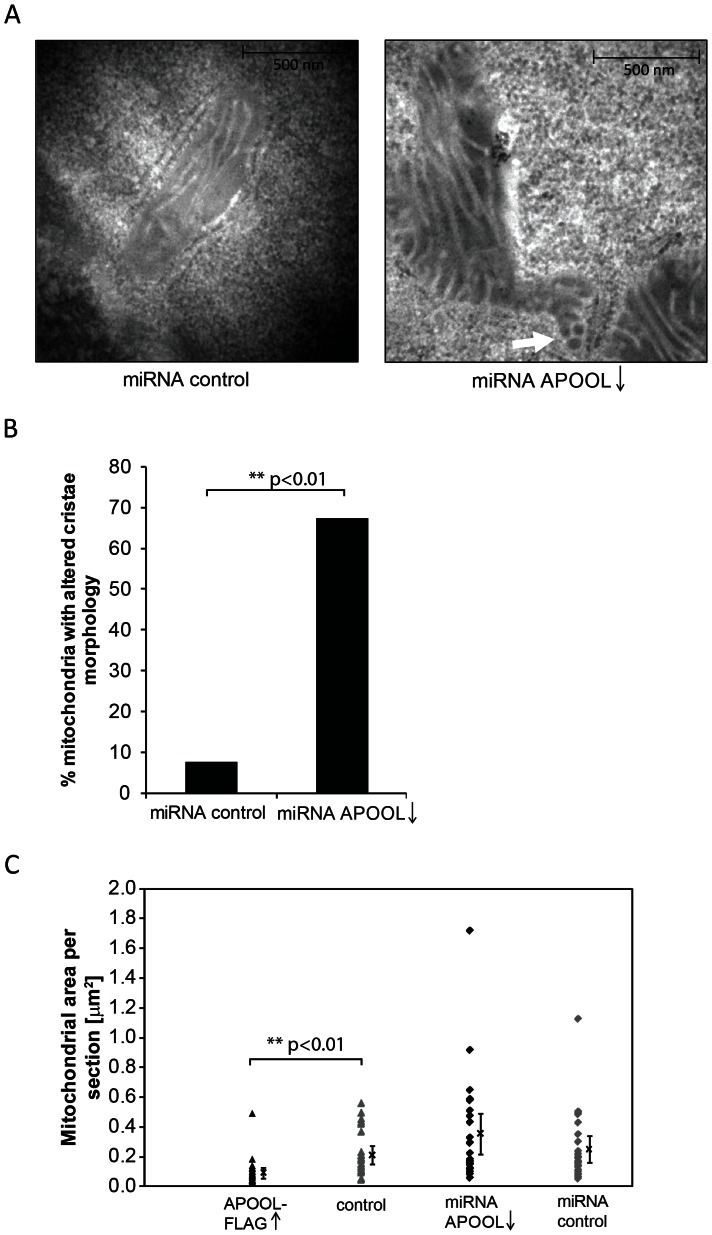
Downregulation of APOOL alters cristae morphology. **A**, Electron micrographs of 143B cells after transfection with plasmid DNA encoding miRNA against APOOL or control miRNA. White arrows indicate concentric, interconnected and/or branched cristae. **B**, Quantification of mitochondrial ultrastructure. The frequency of mitochondria showing interconnected or branched cristae (white arrow) for APOOL downregulation (n = 55) or control (n = 13) were determined. Differences in the amount of mitochondria showing altered cristae morphology were statistically assessed using contingency table analysis followed by the likelihood-ratio test. **C**, Quantification of mitochondrial area per mitochondrial section in electron micrographs either when APOOL was downregulated or when APOOL-FLAG was overexpressed. Corresponding controls for each experiment are indicated. Values for all individual mitochondrial sections analyzed (n = 25 to 26) are indicated on the left of each panel. The mean value ± confidence value at p = 0.95 is indicated to the right of each panel.

APOOL was downregulated by expression of miRNA targeted against APOOL transcripts. The high efficiency of the knockdown was confirmed by Western blot analysis (Fig. 6A, left panel). Downregulation of APOOL did not appear to affect the protein levels of Mitofilin, mtHSP60, OPA1, or DRP1 in total cell extracts (Fig. 6A). OPA1 processing was only moderately increased upon downregulation of APOOL (Fig. 6A, middle panel). Downregulation of APOOL did not lead to a significant increase in ROS formation neither when compared to the corresponding miRNA control nor to untreated cells (Fig. 6B). Only starvation (HBSS) led to increased ROS formation consistent with earlier reports [44], [45]. Mitochondrial morphology was not significantly altered (Fig. 6CD) suggesting that OPA1 processing was not sufficiently altered to impair mitochondrial dynamics. Next we determined the basal mitochondrial OCR and the extent to which this rate is increased by dissipating the mitochondrial membrane potential. HeLa cells with reduced levels of APOOL showed a grossly reduced basal OCR. Moreover, after applying the uncoupler FCCP the OCR was significantly less induced compared to control cells. This clearly shows that downregulation of APOOL has a major influence on mitochondrial respiration. Using electron microscopy we observed that downregulation of APOOL further had a gross impact on mitochondrial ultrastructure (Fig. 7A). The frequency of mitochondrial sections showing cristae with small concentric structures that appeared branched and interconnected was significantly increased upon downregulation of APOOL (Fig. 7B). We further determined whether the average area of mitochondrial sections was increased upon downregulation of APOOL since this was reported for the deletion of Fcj1 in baker's yeast [15], [19]. Downregulation of APOOL appeared to lead to a minor increase in the average area of mitochondrial sections; however, this increase was not statistically significant (Fig. 7C). In contrast, overexpression of APOOL-FLAG led to a significant reduction of the average area of mitochondrial sections consistent with the observed mitochondrial fragmentation (Fig. 4CD). Taken together, these observations strongly suggest an important function for APOOL in maintaining the structural integrity of mitochondria.

### APOOL physically interacts with Mitofilin

**Figure 8:**
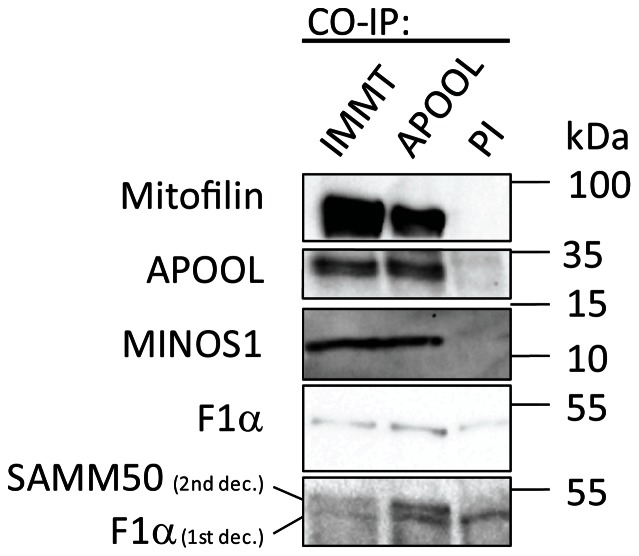
APOOL is a novel subunit of the Mitofilin/MINOS protein complex. Co-immunoprecipitation experiments using indicated antibodies and preimmune serum (PI) were performed. 50% of the elution fractions were loaded and analyzed by western blotting using antibodies raised against indicated proteins. The blot at the bottom was first decorated with antibodies raised against F1α (1st dec.) and subsequently with antibodies raised against SAMM50 (2nd dec.). The first decoration is shown above this western blot result for comparison. Co-IP, antibody used for co-immunoprecipitation; WB, antibody used for detection by western blot; PI, preimmune serum.

Given that APOOL appears to be in the Mitofilin/MINOS complex (Fig. 1) and plays a role in determining cristae morphology (Fig. 5 and 7) we tested whether APOOL physically interacts with subunits of the Mitofilin/MINOS complex. Mitochondria from HeLa cells were solubilized with digitonin and coimmunoprecipation was performed using antibodies raised against Mitofilin, against APOOL, and preimmune serum as control. These experiments revealed that APOOL is co-purified with Mitofilin, MINOS1, and SAMM50 (Fig. 8). In a reciprocal manner, we further could show that Mitofilin co-purifies with APOOL, MINOS1, and SAMM50. In contrast, the mitochondrial subunit F1α of the F_1_F_O_ ATP synthase did not show a specific interaction with APOOL or Mitofilin confirming the specificity of the observed protein-protein interactions. The weak bands corresponding to F1α are present in all three elution fractions including when preimmune serum was used. The fact that for F1α signals were detected at all can be attributed to the high abundance of this subunit in mitochondria and to the high sensitivity of the antibody used for detection. Taken together, we conclude that APOOL, Mitofilin, MINOS1, and SAMM50 are part of the same protein complex and that APOOL indeed is a novel subunit of the Mitofilin/MINOS complex in mammalian cells.

## Discussion

The Mitofilin/MINOS protein complex determines cristae morphology in lower and higher eukaryotes. In baker's yeast five novel subunits of the Fcj1 complex were recently identified and the complex was termed MINOS [16], [17] or MitOS complex [18]. The knowledge about the mammalian complex is still rather limited albeit several interaction partners with Mitofilin have been reported [16], [21], [22], [23], [24], [25]. Using a newly established proteomic complexome profiling approach [37] we were able to reveal two novel putative constituents of this protein complex in its native state, namely APOO and APOOL. Focusing on APOOL we could demonstrate that APOOL interacts with Mitofilin, MINOS1, and SAMM50, confirming that APOOL is indeed part of the Mitofilin/MINOS complex. This also corroborates the power of the complexome profiling method which was described only recently [37]. We further showed that APOOL is a membrane protein exposing major parts to the intermembrane space of mitochondria. This is consistent with our bioinformatic analysis and data from baker's yeast demonstrating that both putative orthologs of APOO/APOOL, Aim37 and Mio27, are membrane-bound proteins also exposed to the IMS [17], [18]. This appears to be in contrast to findings on MOMA-1, the putative homologue of APOO/APOOL in *C. elegans*, which has been suggested to be located in the OM [28]. Still, this was not unambiguously shown as in this study a significant subfraction of MOMA-1 was also found at the IM [28]. Overall several lines of evidence rather point to a location of major parts of APOOL and its orthologs to the IMS of mitochondria. This is also supported by the known location of Mitofilin and Fcj1 in the IMS [13], [15], [46] shown to interact with APOOL as demonstrated here or its orthologs in baker's yeast, respectively [17], [18]. Based on our and the data of others we are not able to determine whether human APOOL is associated with the IM or the OM or with both of these membranes. Even the observed specific binding to cardiolipin (CL) does not help to resolve this matter as CL has been reported to be present in both membranes [47], [48] and to be enriched at contact sites between IM and OM [49]. The latter is interesting as the FCJ1/MINOS complex was shown earlier to be enriched at contact sites [15] and to be anchored to the outer membrane via a physical interaction of the C-terminus of Fcj1 to the TOB55/SAM50 complex [19].

The fact that APOOL is a constituent of the Mitofilin/MINOS complex together with the ability of APOOL to bind CL raises the question on the functional role of this specific lipid binding property. One possibility is that APOOL is involved in the biogenesis or transport of CL (within or between the IM and the OM) and thus affects mitochondrial ultrastructure. Possibly the latter effect is linked to the observation that CL is required for the supramolecular organization of the F_1_F_O_ ATP synthase in mitochondria and cristae morphology [50]. The oligomerization of the F_1_F_O_ ATP synthase is well known to affect cristae morphology [51], [52], [53], [54]. An interesting point to note is that Fcj1 in baker's yeast was shown to impair oligomerization of F_1_F_O_ ATP synthase and acts in an antagonistic manner to subunit e/g of the F_1_F_O_ ATP synthase [15]. In a mouse model for human Barth syndrome which is characterized by low tetralinoleoyl CL levels mitochondrial morphology as well as cristae ultrastructure were greatly distorted [55]. Cristae alterations were further observed in *C. elegans* when CL synthesis is impaired [56]. Taken together, APOOL may well be required for proper transport of CL e.g. to regulate oligomerization of the F_1_F_O_ ATP synthase. The same could be true for assembly of complex IV which was also shown to partly depend on CL [57], [58]. An alternative possibility is that CL is required for efficient assembly of the Mitofilin/MINOS complex itself. Future studies are required to test whether APOOL indeed affects cardiolipin levels or the supramolecular organization of the F_1_F_O_ ATP synthase and whether CL levels influence the assembly of the Mitofilin/MINOS complex. Still, our data indicate that APOOL is linked to the role of CL in determining cristae morphology and it will be interesting to decipher the complex interplay between Mitofilin, MINOS1, APOOL, SAMM50, the F_1_F_O_ ATP synthase and CL.

Next to APOOL which was characterized in detail here we also suggest that APOO is part of the Mitofilin/MINOS complex. This is based on the fact that APOO shows a very unique size distribution in native large pore gel electrophoresis with distinct maxima which resemble very much the profile of the Mitofilin/MINOS complex. Using HeLa cells we could confirm that APOO is indeed a mitochondrial protein (Fig. 1 and data not shown) which is remarkable given that APOO was reported to be a secreted glycoprotein [26]. However, whether APOO is indeed a physical constituent of this complex is part of an ongoing study.

The molecular mechanism of how APOOL influences mitochondrial ultrastructure is currently unclear. It could be linked to regulate CL biogenesis or transport as discussed above. Alternatively, it might affect mitochondrial protein import as this has been reported for the Fcj1/MINOS complex in baker's yeast as well as for Mitofilin/MINOS complex in mammalian cells, respectively [17], [19], [23], [59]. Still, the yeast orthologs of APOOL, Aim37 and Mio27, were shown to have little effect on beta barrel biogenesis [59] making such a role for APOOL at least less likely. Further studies are necessary to study the potential role of APOOL in protein import.

These data also have another unexpected implication as we suggest that the role of apolipoproteins might not be limited to their classical role in lipid transport in the circulatory and lymphatic system but rather also fulfill tasks inside cells and even within organelles. Certainly, our observations do not exclude that APOO or APOOL might be secreted like other apolipoproteins by certain cell types. However, we clearly demonstrate an intracellular and intraorganellar role for APOOL. We suggest the same to be true for APOO. In line with this view, a recent study reported that APOO has no impact on those aforementioned processes apolipoproteins are normally associated with [27].

In summary, we were able to characterize APOOL as a mitochondrial membrane protein which is part of the Mitofilin/MINOS protein complex. The identification of APOOL as a cardiolipin-binding subunit of this complex is an important step in better understanding its role in determining mitochondrial cristae morphology and will provide a basis for further research on the interplay between lipids and protein complexes determining cristae morphology.
